# Supplementation of Lysine and Methionine in Milk Replacer or Starter Concentrate for Dairy Calves in Step-Up/Step-Down Feeding Program

**DOI:** 10.3390/ani11102854

**Published:** 2021-09-29

**Authors:** Jackeline Thais Silva, Evangelina Miqueo, Thaís Manzoni Torrezan, Nathalia Brito Rocha, Giovana Simão Slanzon, Gercino Ferreira Virginio Júnior, Carla Maris Machado Bittar

**Affiliations:** Department of Animal Science, Luiz de Queiroz College of Agriculture, University of São Paulo, Av. Pádua Dias, 11. Piracicaba, São Paulo 13418-900, Brazil; jtsilva@ymail.com (J.T.S.); evangelina.miqueo@gmail.com (E.M.); manzoni.thais@gmail.com (T.M.T.); n.britorocha@yahoo.com.br (N.B.R.); giovana.slanzon@gmail.com (G.S.S.); gercino.ferreiravj@yahoo.com.br (G.F.V.J.)

**Keywords:** amino acids, blood metabolites, calf

## Abstract

**Simple Summary:**

Young animals may have limited growth when amino acid (AA) requirements, especially lysine (Lys) and methionine (Met), are not met. However, the requirements of these AA are not well established for dairy calves, and supplementation strategies may vary with the milk feeding program. This study aimed to evaluate supplementation with Lys and Met in a milk replacer (MR) or a starter concentrate (SC) for calves in a step-up/step-down feeding program. Supplementation with Lys and Met did not benefit the dairy calves’ performance or metabolism when fed higher volumes of MR. Further studies are needed to understand the negative effects of AA on calf starter intake.

**Abstract:**

This study aimed to evaluate the performance and metabolic changes in dairy calves fed in a step-up/step-down program and supplemented with lysine and methionine in a milk replacer (MR) or starter concentrate (SC). Male Holstein calves (*n* = 45) were blocked and distributed in the control without supplementation (1) and with lysine and methionine supplementation in the SC to achieve an intake of 17 and 5.3 g/d, respectively (2), and in the MR to achieve the same daily intake (3). MR was fed 4 L/d until the 2nd week, 8 L/d from the 3rd to 6th week, and 4 L/d from the 7th to 8th week, when calves were weaned. The calves were followed until the 10th week of age. Feed intake was measured daily. Weight and body measurements were registered weekly, and blood samples were collected biweekly. The lysine and methionine intake during the whole period was higher when supplementation occurred via MR. There was a supplementation effect for average daily gain after weaning, and the animals supplemented in the MR had lower BW than those that were not supplemented. Supplementation in MR decreased starter intake at the 10th week and total intake (g DM/d) after weaning. Supplementation with lysine and methionine in the MR or the SC did not benefit the performance or metabolism of dairy calves in the step-up/step-down program. Further studies are needed to understand the effects of amino acid supplementation on feed intake.

## 1. Introduction

The nutritional management of dairy calves can have an impact on the entire animal’s productive life [1]. Low preweaning weight gain can delay age at first calving and further decrease milk production [1,2]. Providing larger liquid diet volumes is one strategy to increase weight gain in the preweaning period, thereby improving the animal’s performance and future milk production [1]. A disadvantage of this management is to reduce the starter concentrate intake (SC), as it is inversely proportional to the liquid diet intake [3]. Thus, weaning can be even more challenging and may decrease performance in the postweaning period.

The step-up/step-down feeding system (SUSD) can be a strategy to mitigate this problem, as the feeding volume is reduced a few days before weaning, as an attempt to stimulate SC intake. This method adjusts the initial feeding volume for young calves with a smaller intake capacity, followed by increased volumes to benefit performance because of the higher growth efficiency. However, at the end of the period, it prepares the calf for an improved weaning process, with the reduction in the feeding volume resulting in increased starter intake. According to Khan et al. [4], a gradual milk volume feeding reduction until weaning increased the concentrate intake compared to calves fed a constant volume (0.851 vs. 0.611 kg).

Another concern besides the liquid diet volume consumed is the nutritional diet quality needed to ensure adequate performance. Dairy calves fed diets formulated solely on the basis of crude protein and energy requirements may not be sufficient to achieve maximum performance of the animals. This is particularly important as the ingredients used for the diet formulation may not meet the amino acid (AA) requirements [5]. Among all AA, Lys and Met are considered essential and the most limiting for growth [6,7,8,9]. The AA requirements have not been determined for dairy calves. However, the recommendation proposed by Hill et al. [8] of 17 g of Lys and 5.3 g of Met intake for calves under 5 weeks of age proved to be adequate, however, for calves fed moderate volumes of milk replacer from 24% to 28% of CP from milk sources.

Several studies have been conducted to establish AA requirements based on the growth rate [8,10,11,12]. According to Gerrits [13], the inclusion of a single AA significantly improves the average daily gain, as also shown by Hill et al. [8] with the inclusion of lysine (Lys) and by Chagas et al. [14] with methionine (Met) addition. However, Silva et al. [15] did not observe a positive effect on performance, gut health, or metabolism, as a result of lysine and methionine supplementation in the milk replacer in association with 0.6% or 1% AminoGut (glutamate and glutamine). These authors concluded that implementing practices to improve starter intake is more important than the fine-tuning of AA. Silva et al. [16], in a subsequent study, showed that supplementing AA through the liquid diet is a more accurate method to achieve daily intake compared to supplementation via SC, because liquid diet intake is more predictable than SC intake.

Bittar et al. [5] analyzed different commercial milk replacer samples and observed lower AA content compared to whole milk. The authors simulated the intake of 4 and 6 L/day, at 12.5% solid dilution, and in both feeding programs, the animals never reached the AA intake values indicated by Hill et al. [8]. Therefore, MR supplementation with synthetic amino acids would be essential to improve calf performance.

Another alternative to meet the calves’ requirements is AA supplementation in SC. However, even on increasing SC intake, fermentative activity is still low. Thus, supplemented AA would reach the intestine in a greater quantity, along with the microbial protein [17].

This study aimed to evaluate the effect of adding Lys and Met to the MR or the SC on the performance and metabolism of dairy calves fed according to an SUSD feeding program. We hypothesize that lysine and methionine supplementation, either in the MR or in the SC, of calves in a step-up/step-down feeding system may benefit performance. Supplementation via MR would be more efficient, while for older calves, supplementation via SC could also benefit performance as solid feed intake increases and rumen develops.

## 2. Materials and Methods

This study was conducted at the calf facilities of the Department of Animal Science, “Luiz de Queiroz” College of Agriculture, University of São Paulo, located in Piracicaba—São Paulo, Brazil. The trial period was from April to August 2013. During this period, the average temperature was 20.3 °C, with a maximum of 27.0 °C and a minimum of 13.5 °C. The mean relative humidity during the study period was 78%, and the average rainfall was 90 mm/mo.

Holstein male calves (*n* = 45) from a commercial farm (2 ± 2 d of age) were blocked considering the birth date (calves within the block presented a difference of no more than 10 d) and weight (37.8 ± 1.08 kg). The calves were separated from the dam at birth, weighed, and fed 2 L of unpasteurized high-quality colostrum (>50 g/L of IgG) in the first two feedings by bottle, providing an intake of 4 L within 10 h of age. If fresh colostrum was not adequate in quality or volume, frozen non-pooled stored colostrum was thawed for the newborn. Only calves with serum protein above 5.5 g/dL at 48 h of life were included in the study [18].

The calves were transported to the experimental calf facility at the Luiz de Queiroz College of Agriculture, University of São Paulo (approximately 100 km), and housed in individual wood shelters (1.35 m height, 1 m width, and 1.45 m depth) distributed in a trimmed grassy field. The calves were contained with a chain belt attached to a thin chain, allowing an adequate walking area but no physical contact with other calves. The individual housing allowed individual feed intake.

The animals had free access to water and a commercial finely ground SC during the entire experimental period (Bezerra Avant Feed, Agroceres Multimix Animal Nutrition Ltd., Rio Claro, SP, Brazil). All calves were bucket-fed with MR (Violet Sprayfo^®^, Sloten do Brasil Ltd., São Paulo, Brazil), beginning with 4 L/d until the 2nd week of life (487 g DM/d), 8 L/d from the 3rd to 6th week of life (974 g DM/d), 4 L/d from the 7th to 8th week of life, when the calves were abruptly weaned. The volume fed was divided into two meals (7:00 and 17:00). The medium CP milk replacer was chosen since it was most widely adopted by dairy farmers at the time of the study, suggesting an opportunity to use AA supplementation for this type of commercial product. The feed intake was measured daily by weighing the feed offered and orts.

The calves were equally and randomly assigned to one of the following treatments: (1) control: milk replacer (MR) and starter concentrate (SC) without amino acid supplementation (*n* = 15); (2) SC Lys:Met: control MR and ground SC with the addition of Lys and Met (*n* = 15); (3) MR Lys:Met: milk replacer with the addition of Lys and Met and the control SC (*n* = 15). Lysine and methionine addition was considered to achieve an intake of 17 and 5.3 g/d, respectively, based on the correction of the composition analysis of MR or SC and the expected feed intake.

For the determination of the AA profile present in the commercial MR and SC, the NIRS calibration curves for dairy products and for concentrates validated by Adisseo Brasil Nutrição Animal Ltd. were used. For calculation, a total intake of 4 L/d (487 g DM/d) or 8 L/d (973 g DM/d) of the MR and an average of 800 g of SC intake during the entire period were considered. Milk replacer evaluation showed a concentration of 1.09 g/100 g DM of Lys and 0.306 g/100 g DM of Met. When the volume fed was 4 L/d of MR, the inclusion was 24.1 g L-lysine (AjiLys^®^ 99, Ajinomoto do Brasil Ind. e Com. de Alimentos Ltd., Pederneiras, Brazil) and 7.85 g DL-methionine (Rhodimet, Adisseo) for each kg of MR. When the supply was 8 L/d of MR, 6.6 g L-lysine (AjiLys^®^ 99, Ajinomoto do Brasil Ind. e Com. de Alimentos Ltd., Pederneiras, Brazil) and 2.35 g of DL-methionine (Rhodimet, Adisseo) for each kg of MR. Starter concentrate analysis resulted in a concentration of 1.40 g/100 g DM of Lys and 0.50 g/100 g DM of Met, leading to a total intake of 11.2 g/d of Lys and 4.0 g/d of Met, considering a planned average intake of 800 g/d of concentrate. The SC was supplemented by adding 7.25 g L-lysine (AjiLys^®^ 99, Ajinomoto do Brasil Ind. e Com. de Alimentos Ltd., Pederneiras, Brazil) and 1.63 g DL-methionine (Rhodimet, Adisseo) for each kg of SC. The desirable amounts of Lys and Met were mixed in a feed mixer.

The SC and MR samples were collected during the experimental period for chemical composition analysis (Table 1). The feed samples were dried (MA035—Marconi, Piracicaba, São Paulo, Brazil) at 55 °C for 72 h and ground to 1 mm in a Wiley mill (Marconi, Piracicaba, Brazil). The samples were dried in an oven at 105 °C for 24 h for dry matter (DM) determination and incinerated in a muffle at 550 °C for 4 h for the determination of crude ash [19]. The total nitrogen concentration was determined using the Leco TruMac^®^ N apparatus (Leco Corporation, St. Joseph, MI, USA) [19], and the values were multiplied by 6.25 to obtain the crude protein (CP) value for each sample. The ether extract (EE) concentration was determined according to the AOAC [19]. Gross energy (GE) was determined by bomb calorimetry PARR 1261 (Parr Instrument Company, Moline, IL, USA).

Weekly before morning feeding, the calves were weighed on mechanical scales and growth measures were taken using a ruler graduated in centimeters for hip width and withers height, and a flexible tape, also graduated in centimeters, for heart girth. All parameters were measured until the 10th week of age, when the study ended. The average daily gain (ADG) and feed efficiency (FE; kg of ADG/kg of total DMI) were calculated for the preweaning (0–56 d) and postweaning (56–70 d) period. The fecal score was monitored daily, and values were given with regard to the fecal fluidity: (1) normal and firm, (2) loose but generally healthy aspect, (3) very loose, non-watery separation, and (4) watery, as described by Larson et al. [20]. If the fecal score was greater than 2, the calves received an oral electrolyte solution for rehydration. Health problems were monitored daily and treated according to veterinary recommendations.

For the evaluation of metabolic parameters, blood samples were always collected two hours after morning feeding during weeks 2, 4, 6, 8, and 10. The blood sample was collected by jugular venipuncture in two vacuum tubes (Vacuette do Brasil, Campinas, Brazil), one containing sodium fluoride and potassium EDTA and the other containing a clot activator. The samples were centrifuged at 2000× *g* (Universal 320R, Hettich, Tuttlingen, Germany) for 20 min at 4 °C. The plasma and serum obtained after centrifugation were freezer-stored (−26 °C) until subsequent analysis. To analyze plasma glucose, total serum protein (TSP), serum albumin, alkaline phosphatase, and serum creatinine, specific commercial enzymatic kits were used (Labtest Diagnóstica S.A., Lagoa Santa, Brazil). β-Hydroxybutyrate (BHB) was measured using a commercial kit, RANBUT (Randox Laboratories, Life Sciences Ltd., Crumlin, UK). Plasma urea nitrogen (PUN) was measured according to the colorimetric method [21], adjusted for reading in the Automatic System for Biochemistry—Model SBA 200 (CELM, Barueri, Brazil).

The experimental design was a randomized block design, with the animals allocated the blocks according to birth weight and age. All data were tested for normal distribution by the Shapiro–Wilk test, and variance homogeneity by the Levene test. The data were analyzed using the MIXED procedure of the SAS statistical package (version 9.4; SAS Institute Inc., Cary, NC, USA). Collection days were considered as repeated measures (model 1). The best covariance structure was identified from different covariance structures by comparing the AICC statistic (Akaike’ information criteria, corrected). The differences were considered significant at *p* < 0.05 unless otherwise stated. Treatment comparison was performed according to the adjusted Tukey test.

$$\mathrm{Model}\;(1):\;Y_{ijk\;}=\mu +T_{i}+I_{j}+TI_{ij}+B_{k}+e_{ijk}$$

where *Y_ijk_* = dependent variable; *μ* = overall average; *T_i_* = treatment effect; *I_j_* = effect of age; *TI_ij_* = treatment–age interaction; *B_k_* = block effect; and *e_ijk_* = experimental error.

## 3. Results

The SC intake increased during the preweaning period (*p* < 0.001), but there was no difference among treatments in this period or at weaning (8th week; Supplementary Figure S1). However, in the 10th week, there was a treatment effect (*p* = 0.01), with greater intake for calves supplemented in the SC (Table 2). The total DMI was also not affected by supplementation in the preweaning period. However, after weaning, animals fed with supplemented MR had a lower total DMI (*p* = 0.01). There was an effect of age *(p* < 0.001) on preweaning and postweaning total DMI, but the interaction between supplementation and age was not significant (Table 2). The lysine and methionine intake during the whole period was affected by the treatments and was higher in milk replacer supplementation and lower in the control group *(p* < 0.001). The intake of both amino acids was also influenced by age and treatment–age interaction *(p* < 0.001; Table 2; Supplementary Figure S2).

There was no effect of supplementation or interaction of supplementation with the animals’ age on body weight (BW) during weaning (8th week), at the end of the trial (10th week), or for the average during the whole study period. However, a significant effect of age was observed (*p* < 0.001) with increasing values over the evaluated weeks (Table 2). Supplementation did not affect average daily gain (ADG) during the preweaning period; however, after weaning, calves fed with supplemented SC had a greater ADG compared to those fed with supplemented MR (*p* = 0.03), but without any difference in BW in the 10th week among animals under the different treatments (Table 2). When considering the ADG for the whole period, ADG was also higher for calves supplemented with Lys and Met via SC compared to those supplemented via MR (*p* = 0.03). The effect of age was observed on ADG pre- and postweaning (*p* < 0.001), but no interaction between age and supplementation occurred.

Supplementation did not affect the average values of hip width (22.8 ± 0.18), withers height (81.3 ± 0.43), and heart girth (81.4 ± 0.60), but there was a significant age effect (*p* < 0.001) with increasing age of the animal. Gains of corporal measures were also not affected by AA supplementation or the interaction between supplementation and animal age, but heart girth and hip width gains increased as calves aged (*p* < 0.001; Table 2)

Amino acid supplementation did not affect feed efficiency (FE; Table 2). However, FE increased with the age of the animals in the preweaning period (*p* < 0.001) and had a significant interaction effect of supplementation with the postweaning age (*p* = 0.05). Just after weaning (9th week), calves supplemented with MR showed a lower FE; however, in the 10th week, all groups showed similar values.

**Figure 1:**
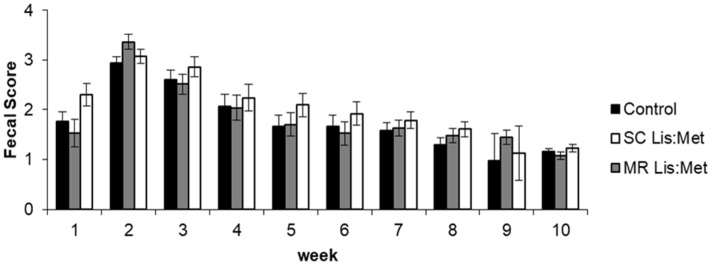
The fecal score of dairy calves in the step-up/step-down feeding system with or without lysine and methionine supplementation in starter concentrate or milk replacer.

The fecal score was not affected by supplementation with Lys and Met (*p* > 0.05), and there was no effect of the interaction between supplementation and age of the animals for this variable (*p* > 0.05; Figure 1). However, there was an effect of age on the fecal score (*p* < 0.001), with higher scores during weeks 2 and 3 of age, commonly defined as the period of higher occurrence of diarrhea.

The blood metabolites evaluated were not affected by supplementation, and there was no effect of the treatment–age interaction. However, there was a significant effect of age (*p* < 0.001), with changes in concentrations over the weeks for all the evaluated metabolites (Table 3).

## 4. Discussion

Despite the supplementation route effect, the animals had similar low intakes until weaning. Quigley [22] recommends weaning once the calf consumes about 680 to 700 g/day of SC, so as to not negatively affect the calf’s performance. However, calves fed a large volume of liquid diet have lower SC intake as liquid and diet solid intakes are negatively correlated [23]. In our study, low SC intake at weaning resulted in a poor performance. Although SC intake increased after reducing the liquid diet feeding volume, this increase was not enough to reach the recommended intake for weaning, which impacted daily gain postweaning, especially for calves supplemented with MR. As the intake of SC was low at the time of weaning, the BHB concentration was also low (Supplementary Figure S1), indicating that the calves were not anatomically and physiologically prepared for weaning to occur. On the other hand, after weaning, the SC intake as well as BHB concentration increased rapidly. This result indicates that the animals quickly adapted to the new diet postweaning, promoting higher ADG in this period compared to preweaning.

After weaning, there was an increase in BW, as a response to increased SC intake and ADG. The calves’ BW at weaning in our study is lower compared to other studies with a supply of larger amounts of liquid diet [24,25,26,27]. The expected BW gain during the preweaning period in a conventional feeding system is approximately 350 g/d [28], whereas in systems providing a more liquid diet, as in this study, ADG should be around 600 g/d, as observed in many studies. In the study by Rincker et al. [26], calves under the intensive feeding system had an ADG of 640 g, but were fed MR with 30.6% CP and 16.1% EE. Omidi-Mirzaei et al. [29] also observed greater ADG in calves in the SUSD feeding system. We speculate that the milk replacer was not adequate for this type of feeding program, as concluded by De Paula et al. [25] when evaluating different feeding programs using the same MR.

Besides the liquid volume fed, other factors negatively affected SC intake in the present study. Silva et al. [15] evaluated Lys and Met supplementation in addition to glutamate and glutamine under similar experimental conditions and observed low starter intake for calves fed 6 L/d during the whole period. In another study by Silva et al. [16], the supplementation of Lys and Met through SC or MR for calves fed 6L/d was also reduced compared to control calves, with a strong reduction for those supplemented through SC. It is important to add that Silva et al. [16] concluded that the supplementation through SC was not an accurate method because solid diet intake is more variable and less predictable and, as a result, does not guarantee adequate daily intake of AA. Other than that, SC particle size and water quality may have also affected the performance of calves, but unfortunately those were not analyzed.

Although concentrate intake was low and milk replacer feeding was controlled, MR-supplemented calves met Hill et al.’s [8] recommendation for lysine intake during the preweaning period, but methionine intake was a few percent lower than expected. However, intake was higher compared to SC-supplemented calves, supporting the data found by Silva et al. [16], suggesting that supplementation via a milk replacer is more accurate. Quigley et al. [30] observed that calves supplemented with essential AA had a SC intake inversely correlated to the daily intake of Lys. According to Muller and Rodriguez [31], the inclusion of methionine in the diet can reduce intake, as it reduces the palatability of the diet. Even though AA supplementation can be related to low SC intake, mainly because of palatability, this may not respond to all the effects in the present study since control calves also presented a low SC intake. In the study by Hill et al. [8], MR with different protein levels and supplemented with lysine and methionine, among other amino acids, resulted in an even lower SC intake compared to our results during preweaning, but a higher intake postweaning. Hill et al. [32] observed the same results after weaning in calves fed MR supplemented with lysine and methionine.

Despite this higher AA intake in the preweaning phase by the MR-supplemented animals, the low starter concentrate intake affected all treatments, resulting in lower ADG. Hill et al. [8] offered a lower volume of liquid diet than that used in the present study and observed greater ADG in calves fed MR supplemented with Lys and Met compared to the ADG observed in our study. Silva et al. [15] also found no differences in BW and ADG among calves fed the same commercial MR supplemented with Lys and Met associated with glutamate and glutamine. However, in a subsequent study, Silva et al. [16] observed pre- and postweaning lower body weight and a tendency for decreased ADG for calves supplemented with Lys and Met through SC or the same commercial MR. In a study with a liquid diet similar to that used in our study, Hill et al. [33] observed an ADG of 430 g/d, a value approximately twice that of the highest average ADG in the preweaning period observed in our study. We were expecting differences in ADG and weight pre- and postweaning for supplemented calves, with improved performance preweaning for calves supplemented via MR, and postweaning for those supplemented via SC. However, because of the low intake in both periods, there were no positive effects.

Lys and Met supplementation was expected to increase BW, corporal measures, and feed efficiency since it would increase the availability of the most limiting AA for growth. However, we expected that the magnitude of the response would be greater for calves supplemented through MR during the preweaning and through SC during the postweaning phase. Conversely, no differences were observed in body measures, even though a higher SC intake was observed postweaning for calves supplemented via SC. In addition, the final BW was not affected by supplementation.

Recently, Quigley et al. [34] analyzed digestibility data from different studies of preweaning dairy calves and concluded that dry matter, fat, and nitrogen digestibility of the liquid diet reached a maximal point at 20 days of age. This indicates that any digestive disturbances in this period could affect the animals’ performance. As observed in our study, in this same period cited by the authors, all treatments had a high diarrhea incidence, potentially impacting long-term performance. However, it is interesting to note that animals supplemented via MR performed worse than those supplemented via SC, and even worse than control animals, in the long term. This may indicate not only an advantage of concentrate supplementation compared to MR, but also that MR supplementation, no matter how accurate it may be, as suggested by Silva et al. [16], may not be beneficial to diarrheic animals.

The evaluated metabolites were not affected by AA supplementation but by the age of the calves, mainly because of the transition from a pre- to a functional ruminant condition, as a result of increased SC intake as calves aged. Despite AA supplementation and higher lysine and methionine intake by MR-supplemented calves, the blood metabolites that explain protein and AA metabolism, such as TSP, creatinine, albumin, and plasma urea-N, were not affected.

## 5. Conclusions

Lysine and methionine supplementation in a starter concentrate or milk replacer was not beneficial to the performance or metabolism of calves in a step-up/step-down feeding system. Although AA supplementation in milk replacer seems to be more accurate during preweaning, this type of supplementation proved to be inadequate after weaning, since the supply of liquid diet is discontinued. Supplementation via starter concentrate proved to be more beneficial from this point of view, even though it did not reach the desired values during the preweaning period. The effects of AA supplementation on calves’ feed intake need further investigation.

## Figures and Tables

**Table 1:** Chemical composition of milk replacer (with 4 and 8 L/d supply) and starter with or without lysine and methionine supplementation.

	MR	MR LYS:MET 4 L/d	MR LYS:MET 8 L/d	SC	SC LYS:MET
Dry matter (%)	95.72	95.72	95.61	91.13	90.73
Ash (%DM)	8.14	8.11	8.47	6.56	6.40
Crude protein (%DM)	21.57	22.33	21.34	20.50	21.40
Ether extract (%DM)	13.79	14.25	14.45	6.11	5.92
Gross energy (cal/kg)	4360.12	4220.54	4368.68	4072.03	4059.85
Lysine (%)	1.09	3.50	1.75	1.40	2.13
Methionine (%)	0.31	1.09	0.55	0.50	0.66

MR: Milk replacer without lysine and methionine supplementation; MR LYS:MET 4 L/d: milk replacer supplemented with lysine and methionine for the period with the supply of 4 L/d; MR LYS:MET 8 L/d: milk replacer supplemented with lysine and methionine for the period with the supply of 8 L/d; SC: starter concentrate without lysine and methionine supplementation; SC LYS:MET: starter concentrate supplemented with lysine and methionine.

**Table 2:** Starter intake, total intake, and feed efficiency of dairy calves in the step-up/step-down feeding system with or without lysine and methionine supplementation in starter or milk replacer.

	Treatment			SEM	*p*-Value		
	Control	SC Lys:Met	MR Lys:Met		T	A	T × A
**Starter concentrate intake (g/d)**							
Preweaning	71.4	101.0	54.3	18.34	0.22	<0.001	0.42
At weaning *	306.0	348.3	180.2	68.21	0.22	--	--
10th week	1502.7 ^a^	1615.6 ^a^	1235.1 ^b^	94.41	0.01	--	--
**Total DM intake (g/d)**							
Preweaning	793.7	850.2	787.6	20.98	0.08	<0.001	0.08
Postweaning	1293.5 ^a^	1341.3 ^a^	950.6 ^b^	90.69	0.01	<0.001	0.33
**AA Intake during the whole period, g/d**							
Lysine	10.26 ^c^	13.54 ^b^	16.43 ^a^	0.567	<0.001	<0.001	<0.001
Methionine	3.23 ^c^	4.04 ^b^	4.78 ^a^	0.183	<0.001	<0.001	<0.001
**Body weight (kg)**							
Initial	37.6	37.9	38.0	1.08	0.94	--	--
At weaning *	47.4	49.0	47.3	1.25	0.93	--	--
10th week	54.7	58.3	52.9	1.87	0.48	--	--
Whole period	44.0	45.4	44.4	1.09	0.64	<0.001	0.11
**Average daily gain (g)**							
Preweaning	193.9	232.0	151.7	34.44	0.27	<0.001	0.18
Postweaning	553.5 ^ab^	676.1 ^a^	344.0 ^b^	83.70	0.03	0.016	0.08
Whole period	270.6 ^ab^	327.1 ^a^	195.3 ^b^	33.84	0.03	<0.001	0.09
**Corporal measures gain, cm/week**							
Withers height	0.64	0.76	0.65	0.41	0.98	0.447	0.63
Heart girth	1.09	1.44	0.96	0.18	0.18	<0.001	0.70
Hip width	0.33	0.30	0.31	0.03	0.89	<0.001	0.49
Feed efficiency **							
Preweaning	0.18	0.19	0.19	0.04	0.21	<0.001	0.25
Postweaning	0.46	0.50	0.35	0.053	0.12	0.595	0.05

SEM, Standard error of mean; AA, Amino acid; T, treatment effect; A, age effect (week); T × A, treatment–age interaction; Control, a basal diet without supplementation with amino acids; SC Lys:Met, starter concentrate supplemented with lysine and methionine; MR Lys:Met, milk replacer supplemented with lysine and methionine; * weaning occurred during the 8th week of age; ** calculated as average daily gain/total DMI; ^a–c^ mean values with different superscripts within the same parameter are different at *p* < 0.05.

**Table 3:** Blood metabolites of dairy calves in the step-up/step-down feeding system with or without lysine and methionine supplementation in starter or milk replacer.

	Treatment			SEM	*p*-Value		
	Control	SC Lys:Met	MR Lys:Met		T	A	T × A
Glucose (mg/dL)	91.7	90.8	87.2	2.31	0.22	<0.001	0.62
BHBA (mmol/L)	0.09	0.10	0.08	0.01	0.48	<0.001	0.86
Alkaline phosphatase (U/L)	126.9	127.7	103.5	9.93	0.17	<0.001	0.08
Creatinine (mg/dL)	0.83	0.80	0.84	0.03	0.73	<0.001	0.13
Total serum protein (g/dL)	5.6	5.7	5.6	0.09	0.76	<0.001	0.64
Albumin (g/dL)	2.7	2.8	2.8	0.04	0.71	<0.001	0.84
Plasma urea-N (mg/dL)	10.9	10.1	10.4	0.47	0.48	<0.001	0.93

SEM, standard error of mean; T, treatment effect; A, age effect (week); T × A, treatment–age interaction; Control, a basal diet without supplementation with amino acids; SC Lys:Met, starter concentrate supplemented with lysine and methionine; MR Lys:Met, milk replacer supplemented with lysine and methionine.

## Data Availability

The data presented in this study are available on request from the corresponding author. The data are not publicly available due to restrictions by the research group.

## Supplementary Materials

The following are available online at https://www.mdpi.com/article/10.3390/ani11102854/s1, Figure S1: Starter intake and BHBA concentration according to age of dairy calves in step-up/step-down feeding system with or without lysine and methionine supplementation in starter or milk replacer. Age effect of *p* < 0.001 for both starter intake and BHBA; Figure S2: Lysine and Methionine intake according to age of dairy calves in step-up/stepdown feeding system with or without lysine and methionine supplementation in starter or milk replacer. Age effect of and an interaction of treatment and age effect *p* < 0.001 for lysine and methionine intake. * Denotes difference of MR Lys:Met to both Control and SC Lys:Met; ¥ Denotes difference of SC Lys:Met to both Control and MR Lys:Met.

## References

[1] Soberon F., Raffrenato E., Everett R.W., Van Amburgh M.E. (2012). Preweaning milk replacer intake and effects on long-term productivity of dairy calves. J. Dairy Sci..

[2] Bach A., Domingo L., Montoro C., Terré M. (2013). Short communication: Insulin responsiveness is affected by the level of milk replacer offered to young calves. J. Dairy Sci..

[3] Gelsinger S.L., Coblentz W.K., Zanton G.I., Ogden R.K., Akins M.S. (2020). Physiological effects of starter-induced ruminal acidosis in calves before, during, and after weaning. J. Dairy Sci..

[4] Khan M.A., Lee H.J., Lee W.S., Kim H.S., Kim S.B., Ki K.S., Ha J.K., Lee H.G., Choi Y.J. (2007). Pre- and Postweaning Performance of Holstein Female Calves Fed Milk Through Step-Down and Conventional Methods. J. Dairy Sci..

[5] Bittar C.M.M., da Silva J.T., Chester-Jones H. (2018). Macronutrient and amino acids composition of milk replacers for dairy calves. Rev. Bras. Saúde e Produção Anim..

[6] Williams A.P., Hewitt D. (1979). The amino acid requirements of the preruminant calf. Br. J. Nutr..

[7] Tzeng D., Davis C.L. (1980). Amino Acid Nutrition of the Young Calf. Estimation of Methionine and Lysine Requirements. J. Dairy Sci..

[8] Hill T.M., Bateman H.G., Aldrich J.M., Schlotterbeck R.L., Tanan K.G. (2008). Optimal Concentrations of Lysine, Methionine, and Threonine in Milk Replacers for Calves Less than Five Weeks of Age. J. Dairy Sci..

[9] Li H., Diao Q., Zhang N.F., Fan Z.Y. (2008). Growth, Nutrient Utilization and Amino Acid Digestibility of Dairy Calves Fed Milk Replacers Containing Different Amounts of Protein in the Preruminant Period. Asian-Australas. J. Anim. Sci..

[10] Gerrits W.J.J., France J., Dijkstra J., Bosch M.W., Tolman G.H., Tamminga S. (1997). Evaluation of a Model Integrating Protein and Energy Metabolism in Preruminant Calves. J. Nutr..

[11] Hill T.M., Bateman H.G., Aldrich J.M., Schlotterbeck R.L. (2009). Effect of Weaning Age of Dairy Calves Fed a Conventional or More Optimum Milk Replacer Program. Prof. Anim. Sci..

[12] Hill T.M., Bateman H.G., Aldrich J.M., Quigley J.D., Schlotterbeck R.L. (2013). Evaluation of ad libitum acidified milk replacer programs for dairy calves. J. Dairy Sci..

[13] Gerrits W.J.J. (2019). Symposium review: Macronutrient metabolism in the growing calf. J. Dairy Sci..

[14] Chagas J.C.C., Ferreira M.A., Faciola A.P., Machado F.S., Campos M.M., Entjes M.R., Donzele J.L., Marcondes M.I. (2018). Effects of methionine plus cysteine inclusion on performance and body composition of liquid-fed crossbred calves fed a commercial milk replacer and no starter feed. J. Dairy Sci..

[15] da Silva J.T., Manzoni T., Rocha N.B., Santos G., Miqueo E., Slanzon G.S., Bittar C.M.M. (2018). Evaluation of milk replacer supplemented with lysine and methionine in combination with glutamate and glutamine in dairy calves. J. Appl. Anim. Res..

[16] da Silva J.T., Miqueo E., Torrezan T.M., Rocha N.B., Slanzon G.S., Virgínio Júnior G.F., Bittar C.M.M. (2021). Lysine and Methionine Supplementation for Dairy Calves Is More Accurate through the Liquid than the Solid Diet. Animals.

[17] Sancanari J.B.D., Ezequiel J.M.B., Galati R.L., de Figueiredo Vieira P., Seixas J.R.C., Santamaria M., Kronka S.N. (2001). Efeito da metionina protegida e não protegida da degradação ruminal sobre a produção e composição do leite de vacas holandesas. Rev. Bras. Zootec..

[18] Elsohaby I., McClure J.T., Waite L.A., Cameron M., Heider L.C., Keefe G.P. (2019). Using serum and plasma samples to assess failure of transfer of passive immunity in dairy calves. J. Dairy Sci..

[19] AOAC International (2012). Official Methods of Analysis.

[20] Larson L.L., Owen F.G., Albright J.L., Appleman R.D., Lamb R.C., Muller L.D. (1977). Guidelines Toward More Uniformity in Measuring and Reporting Calf Experimental Data. J. Dairy Sci..

[21] Chaney A.L., Marbach E.P. (1962). Modified Reagents for Determination of Urea and Ammonia. Clin. Chem..

[22] Quigley J.D. (1996). Influence of Weaning Method on Growth, Intake, and Selected Blood Metabolites in Jersey Calves. J. Dairy Sci..

[23] Khan M.A., Weary D.M., von Keyserlingk M.A.G. (2011). Invited review: Effects of milk ration on solid feed intake, weaning, and performance in dairy heifers. J. Dairy Sci..

[24] Raeth-Knight M., Chester-Jones H., Hayes S., Linn J., Larson R., Ziegler D., Ziegler B., Broadwater N. (2009). Impact of conventional or intensive milk replacer programs on Holstein heifer performance through six months of age and during first lactation. J. Dairy Sci..

[25] de Paula M.R., Oltramari C.E., Silva J.T., Gallo M.P.C., Mourão G.B., Bittar C.M.M. (2017). Intensive liquid feeding of dairy calves with a medium crude protein milk replacer: Effects on performance, rumen, and blood parameters. J. Dairy Sci..

[26] Davis Rincker L., VandeHaar M.J., Wolf C., Liesman J., Chapin L., Weber Nielsen M. (2011). Effect of intensified feeding of heifer calves on growth, pubertal age, calving age, milk yield, and economics. J. Dairy Sci..

[27] Margerison J.K., Robarts A.D.J., Reynolds G.W. (2013). The effect of increasing the nutrient and amino acid concentration of milk diets on dairy heifer individual feed intake, growth, development, and lactation performance. J. Dairy Sci..

[28] NRC (2001). Nutrient Requirements of Dairy Cattle.

[29] Omidi-Mirzaei H., Khorvash M., Ghorbani G.R., Moshiri B., Mirzaei M., Pezeshki A., Ghaffari M.H. (2015). Effects of the step-up/step-down and step-down milk feeding procedures on the performance, structural growth, and blood metabolites of Holstein dairy calves. J. Dairy Sci..

[30] Quigley J.D., Schwab C.G., Hylton W.E. (1985). Development of Rumen Function in Calves: Nature of Protein Reaching the Abomasum. J. Dairy Sci..

[31] Muller L.D., Rodriguez D. (1975). Methionine Hydroxy Analog Supplementation of Low Protein Calf Rations. J. Dairy Sci..

[32] Hill T.M., Aldrich J.M., Schlotterbeck R.L., Bateman H.G. (2007). Amino Acids, Fatty Acids, and Fat Sources for Calf Milk Replacers. Prof. Anim. Sci..

[33] Hill T.M., Bateman H.G., Aldrich J.M., Schlotterbeck R.L. (2010). Effect of milk replacer program on digestion of nutrients in dairy calves. J. Dairy Sci..

[34] Quigley J.D., Dennis T.S., Suarez-Mena F.X., Hill T.M., Aragona K.M. (2021). Meta-analysis of effects of age on intestinal digestibility of liquid feeds in young calves. JDS Commun..

