# Supplementary material for: Supplementation of Lysine and Methionine in Milk Replacer or Starter Concentrate for Dairy Calves in Step-Up/Step-Down Feeding Program
Source: Animals (Basel). 2021 Sep 29;11(10):2854. doi: 10.3390/ani11102854 (PMC8532968; doi:10.3390/ani11102854)
Supplement: Supplementary file 1 [file animals-11-02854-s001.zip › animals-1365281-supplementary.pdf]

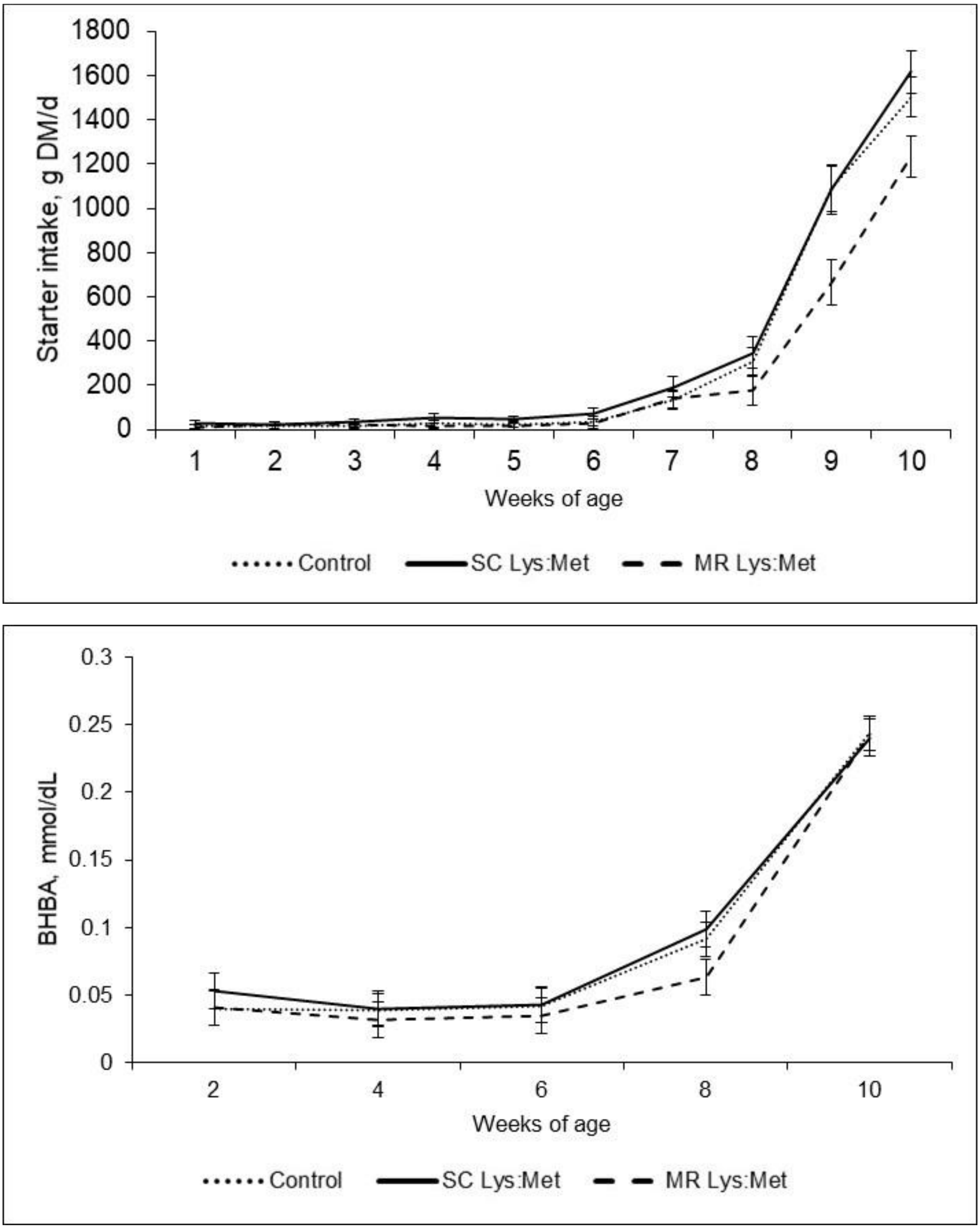

Figure S1. Starter intake and BHBA concentration according to age of dairy calves in step-up/step-down feeding system with or without lysine and methionine supplementation in starter or milk replacer. Age effect of $p < 0.001$ for both starter intake and BHBA concentration.

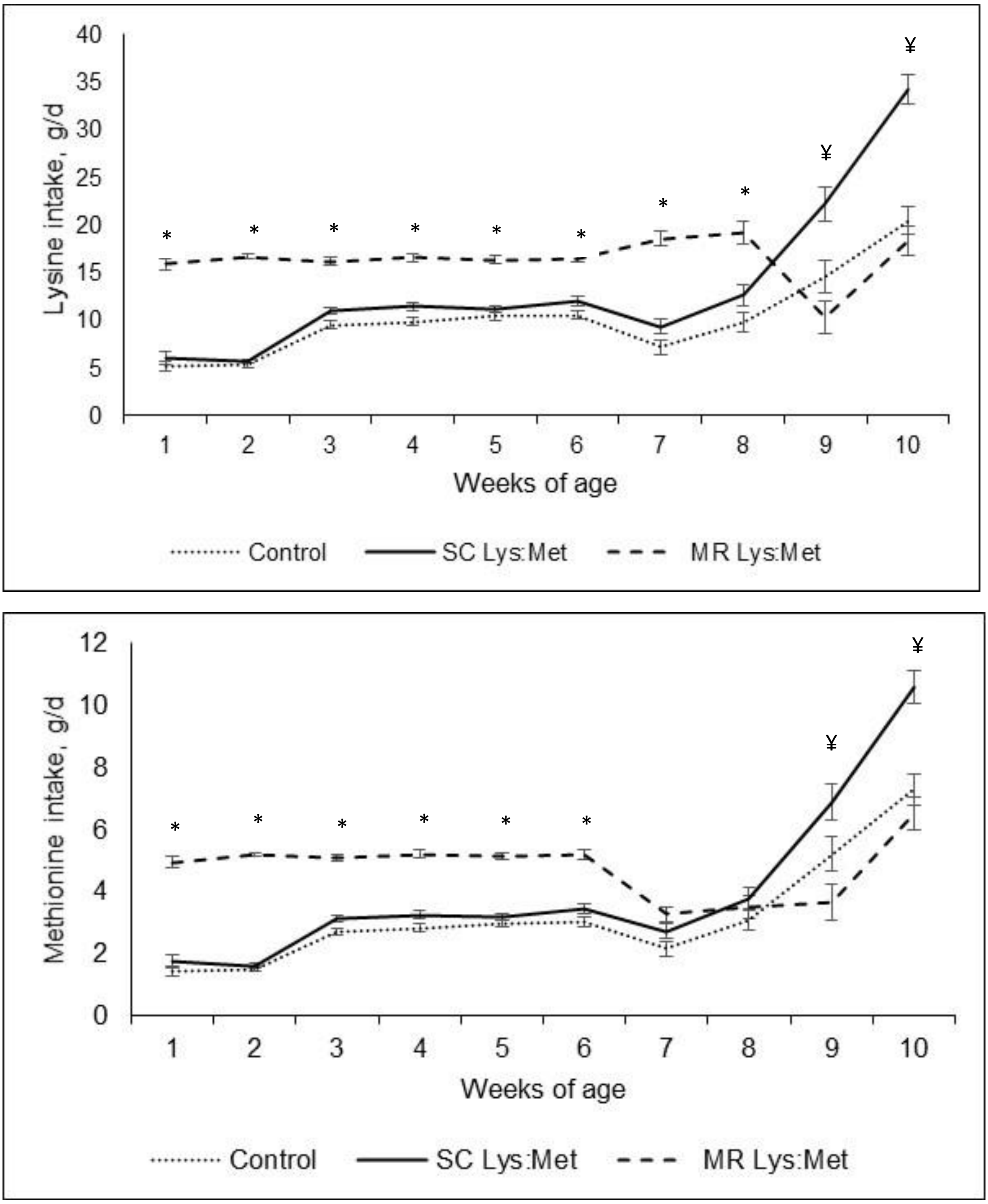

Figure S2. Lysine and Methionine intake according to age of dairy calves in step-up/step-down feeding system with or without lysine and methionine supplementation in starter or milk replacer. Age effect of and an interaction o treatment and age effect $p < 0.001$ for lysine and methionine intake. *Denotes difference of MR Lys:Met to both Control and SC Lys:Met; ¥ Denotes difference of SC Lys:Met to both Control and MR Lys:Met.
